# Treatment of dorsal wrist ganglion cyst by establishing midcarpal volar portal using the “Kiss-in” method

**DOI:** 10.3389/fsurg.2022.944396

**Published:** 2022-08-31

**Authors:** Bo Chen, Leining Wang, Kejiong Liang, Bing Wang, Shuai Jiang, Haifei Shi

**Affiliations:** ^1^ Department of Orthopedics, The First Affiliated Hospital, School of Medicine, Zhejiang University, Hangzhou, China; ^2^ Department of Surgery of Hand and Foot, Beilun People's Hospital, Ningbo, China

**Keywords:** arthroscopic surgery, ganglion cyst, wrist, aesthetics, techniques utilization

## Abstract

**Introduction:**

This paper introduces the treatment and clinical outcome of the dorsal wrist ganglion cyst utilizing the Kiss-in method to establish a midcarpal volar portal.

**Materials and methods:**

Patients with dorsal ganglia of the wrist (*n* = 12, 6 females, 6 males) underwent arthroscopic surgery using the Kiss-in method at our hospital between September 2018 and January 2021. All patients underwent preoperative radiological investigations, such as magnetic resonance imaging (MRI; 12 cases) or ultrasonography (12 cases). The mean age of patients was 30.7 years (range: 19–46 years). The time lost from work, the wrist motion and strength, the presence of scarring, residual symptoms, complications, and recurrence were recorded at a mean follow-up of 24 months.

**Results:**

Eleven patients showed a good prognosis with active motion recovery. One patient showed the recurrence of ganglion, and the second arthroscopic resection was performed 5 months after the first surgery for this patient. After the surgery, the patient fully recovered.

**Conclusions:**

Establishing the midcarpal volar portal by the Kiss-in method is safe. The dorsal ganglion cyst resection through the established midcarpal volar portal is a promising approach, allowing better visualization and a broader range motion of the arthroscope.

## Introduction

Wrist ganglion is a common benign soft tissue mass of the hand, which arises from articular (1, 2) or myxoid degeneration (3). Wrist ganglion accounts for 60%–70% of wrist tumors, being more common in women (4). Most dorsal ganglions appear under the skin between the extensor tendons on the radial side of the dorsal aspect of the carpus. The base of implantation is located in the radiocarpal or in the midcarpal joint, which is in proximity to the scapholunate ligament. Inadequate resection of the ganglions remains the major cause of high recurrences rates, whether it is treated with aspiration or open excision (5). Comparing recurrence rates of open surgery with arthroscopic ganglion resection remains controversial (6). However, with the rapid development of people's financial status, especially female patients, there is an increased requirement for esthetic hand appearance and quality of life. Thus, arthroscopic ganglion resection has become the first choice for women and/or young people (7, 8). Currently, the radial midcarpal portal (MCR) and the ulnar midcarpal portal (MCU) are the commonly used approaches in midcarpal dorsal ganglion resection (9). The scope from the MCU portal has poor vision. Here, we applied a “Kiss-in” method to establish the midcarpal volar portal to treat the dorsal midcarpal ganglia.

## Materials and methods

We reported a series of 12 patients (6 female, 6 male) with dorsal wrist ganglia, who were arthroscopically operated on utilizing the Kiss-in method at our institute between September 2018 and January 2021. All patients underwent preoperative radiological investigations, such as magnetic resonance imaging (MRI; 12 cases) or ultrasonography (12 cases). All patients' cyst base of implantation was on the dorsal scapholunate ligament. The mean age of patients was 30.7 years (range: 19–46 years). The mean surgical procedure duration was 71.4 min (range: 50–115 min). The average length between the appearance of the ganglion and surgical resection was 8 months (range: 3–36 months). The regular indication for arthroscopic resection was the unacceptable open surgery incision scar appearance. All patients had normal mobility, but nine patients experienced pain. Hand strength was measured by the Jamar test, in which four patients showed reduced hand strength with an average loss of 30% compared to the opposite side. The time lost from work, the wrist motion and strength, the presence of scarring, residual symptoms, complications, and recurrence were recorded at a mean follow-up of 24 months. The satisfaction of each patient was also evaluated, as the patient-rated wrist evaluation (PRWE) score for the functional aspect.

### Surgical instruments

A Smith & Nephew small arthroscopy system (2.7 mm, 30°), a traction tower, a shaver, and the like were used during surgery.

### Preparation and patient positioning

The technique used (a Smith & Nephew small arthroscopy system; 2.7 mm, 30°) was the same for all patients. All surgeries were performed by the same surgeon. The surgery was performed as day surgery under brachial plexus nerve anesthesia. The tourniquet applying countertraction was placed on the patient's arm near the elbow to minimize the leverage during upward traction. After exsanguinating and placing an upper limb sterile drape, traction was applied using a traction system or tower. The required traction of 5 kg was applied using the finger traps. The patient lay supine with the shoulder at 90° abduction. The surgeon's position was at the head of the patient, while the assistant's position was at the palmar side of the wrist. The arthroscopy column was usually on the other side of the patient facing the surgeon.

### Dorsal wrist ganglion surgical portal by the Kiss-in method

**Figure 1:**
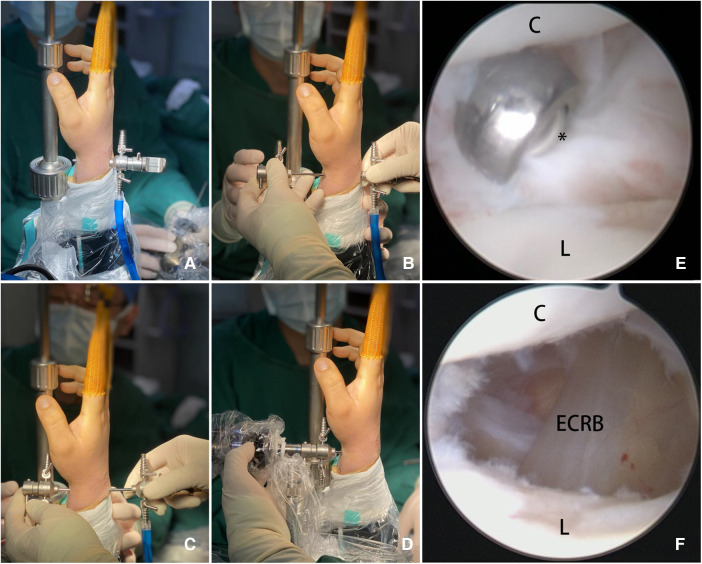
(**A**) Pushing the trocar throughout the Kiss-in point; (**B**) two trocar cannulas and a trocar needle could fit each other and combine into a smooth pipe (Kiss); (**C**) pushing the trocar combination from the volar to dorsal into the joint (in); (**D**) an arthroscope was placed in the volar portal with a better vision: the Kiss-in method was accomplished; (**E**) arthroscopic views of the ganglion stalk excised with a shaver; and (**F**) debridement of the capsule continues distally toward the very dorsal SL joint ligament exposing the ECRB tendons. ECRB, extensor carpi radialis brevis tendon; C, capitate bone; L, lunate bone; *ganglion stalk excised with a shaver.

**Figure 2:**
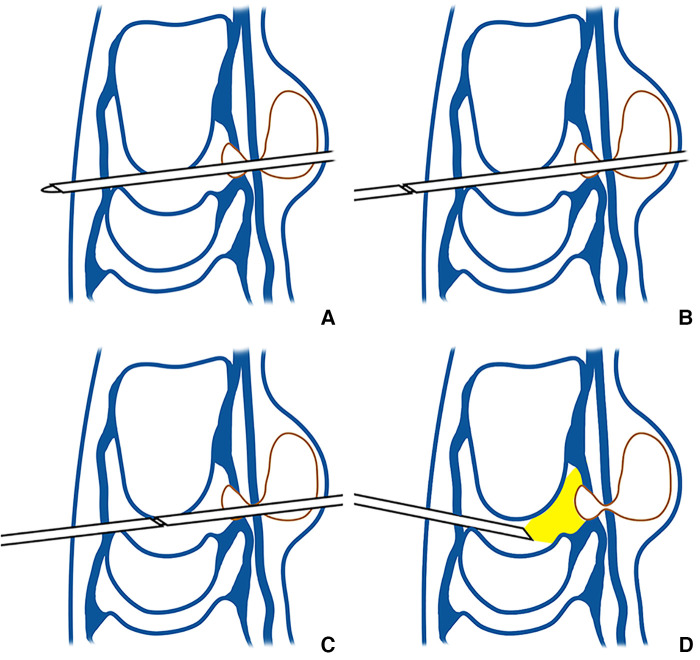
(**A**-**D**) Kiss-in method's sketches for each stage of **Figure 1** (**A**-**D**) in sequence.

First, a small incision was made on the radial midcarpal portal (MCR) or trans-cystic portal. After introducing a blunt trocar, the scope was placed to visualize the intact volar intraarticular portion and a small part of the dorsal view. The arthroscope and the trocar were pushed onto the volar ligament interval under the field of vision and were held still by the operator. The arthroscopic light source was used to locate and mark the Kiss-in point, which was positioned on the surface of the flexor carpi radialis tendon on the palmar skin with methylthionine chloride. A blunt mandrel swop with the trocar was penetrated between the radioscaphocapitate ligament and the long radiolunate ligament, as far as possible to the radial side, and reached the radial side of the flexor carpi radialis tendon, then slid to the ulnar side of the flexor carpi radialis tendon. It could be palpated under the volar skin around the Kiss-in point after pushing through the volar ligament interval and the subcutaneous tissue. A small, 1–2 mm skin incision was made on the Kiss-in point to push the trocar throughout the skin. Another trocar was slipped over the mandrel tip due to the oblique orifice of the trocar cannula. Two trocar cannulas and a trocar needle could fit each other by combining them into a smooth pipe. This process is named KISS. The MCV was established after pushing the trocar combination from the volar to the dorsal into the joint. Then, the Kiss-in method was accomplished (Figures 1A–D, 2), for which the outflow portal was MCU. In brief, the Kiss-in method is that a trocar with no needle kisses into a blunt trocar from the MCR portal through palmar skin, forming a smooth pipe and slowly entering into the midcarpal joint.

The shaver was placed in the dorsal portal, and the arthroscope was placed in the volar portal (Figure 1E). If synovial hyperplasia or damage in the joint was predominant, it was cleaned first. The MCV portal exploration usually reveals a dorsal synovial bulge at the scapholunate interval corresponding to the intraarticular portion of the ganglion or even some outflow of cystic fluid when the external pressure is on the ganglion. The cyst pedicle was shaved by a shaver until visible light yellow or transparent cyst fluid flowed into the joint. At this time, cyst appearance was reduced or even vanished. A part of the joint capsule and cyst wall around the pedicle, generally around 0.5 cm, was removed until the extensor tendons were exposed. The incisions did not require suturing (Figure 1F). Small sterile strips were applied to reduce tension, and a splint was placed for 1 week, followed by the start of rehabilitation.

## Results

### Successes

The origin of the cyst was reconfirmed arthroscopically. Also, synovial membrane cleaning was done on 7 of 12 cases due to the confirmed intra-articular synovial hyperplasia arthroscopically. No other intra-articular pathology was detected. At an average follow-up of 24 months (range: 15–39), 11 out of 12 cases showed an excellent prognosis with active motion recovery. There were no complications, scars, or recurrence. The mean functional recovery time was 9 days, and the average time lost from work was 14 days.

Patients were not reviewed clinically but were questioned by phone or WeChat (a Chinese instant messaging app) after at least 15 months of long-term follow-ups. The PRWE score was calculated for each patient, and the results were as follows: pain score of 6.4/50 and function score of 1.22/50.

### Complications and failures

A complication was observed in one case. The patient continued to perform “heavy manual” work after recovery and was not aware of wrist pain, reduced motion, and cyst recurrence until 4 months later. In this case, incomplete excision of a polypedicle cyst was found during re-examination by an MRI after the first arthroscopic surgery. The second arthroscopic surgery was performed 5 months afterward. Until now, the patient had a fully functional recovery and returned to “heavy manual” work.

## Discussions

The structure of the wrist joint is complex. The carpal bones and the internal and external ligaments are tiny, fragile, and relatively vulnerable. The movement of wrist joints is multidirectional and easy to be damaged. The small space of the wrist joint, especially in the midwrist, is difficult to operate, making the diagnosis and treatment of wrist diseases more difficult.

In recent years, wrist arthroscopy has become an appealing diagnostic approach compared to computed tomography (CT) and MRI, becoming known as the “gold standard.” Like other large arthroscopic techniques, including shoulder joint and knee joint arthroscopy, correct selection and establishment of an arthroscopic wrist portal are conducive to diagnosis and treatment. The accuracy of arthroscopic diagnosis can be improved through multiple approaches, such as repeated comparative observation and comprehensive analysis from different angles.

The dorsal portal is used in most wrist arthroscopic surgeries, whereas the volar portal is relatively rare. Regarding the midcarpal dorsal ganglia, the procedure can be difficult due to the smaller dimension of the joint. Furthermore, it requires a longer surgery length (10). The possible reasons for the lack of volar portal utilization are as follows. First, there are many important structures of the volar wrist, such as blood vessels, nerves, and tendons, and a higher possibility of causing collateral injury for establishing the volar portal. Second, the existing methods of “inside-out” (11) or “outside-in” (12) to establish the volar portal have certain blindness, possibly causing the injury of the volar ligament of the wrist joint.

### Advantages of establishing volar portal using the Kiss-in method

Tham et al. (11) used the “inside-out” method to enter the mandrel located between the long radiolunate ligament (LRL) and the radio-scapho-capitate ligament (RSC). The radial radiocarpal portal was established by penetrating the skin on the ulnar side of the radial flexor carpi tendon, which was relatively close to the median nerve and was likely to cause secondary injuries.

Naroura et al. (13) used the “inside-out” technique of mandrel to establish four volar portals in a cadaveric study. Gillis et al. (14) have verified the possibility of using the trocar pushed from dorsal to volar to establish the volar midcarpal portals by the “inside-out” technique. In clinical practice, puncture of the mandrel must be performed under direct arthroscopy. Consequently, there are three portals and three incisions on the skin.

Unlike the “inside-out” technique of mandrel, the Kiss-in method does not require arthroscopic observation of the mandrel position during the puncture. The cannula is already pressed against the volar ligament interval, which is locating the Kiss-in point inside. Thus, there are two portals/incisions on the skin.

The Kiss-in method can also be applied to other volar portals. In contrast to the “inside-out” technique of the mandrel, the Kiss-in method utilizes the matching orifice between the two cannulas and the connection between the mandrel and the palmar cannula to form a relatively closed and strong tube that is smoother. Moreover, the secondary injury to the wrist can be minimized by retrograde entry. It should be noted that the Kiss-in method requires two trocar cannulas.

It was verified no nerve damage by retrospective data. The determination of Kiss-in points is extremely important. There is a loose connective tissue between the radioscaphocapitate ligament and the long radiolunate ligament that can easily pass through. The radial deviation toward flexor carpi radialis avoids the median and radial nerve. The blunt trocar decreases the probability of nerve injuries. The operation technique varies from person to person, but our skilled and cautious operator did good work.

### Advantages of the midcarpal volar portal for dorsal ganglion resection

In general arthroscopic surgery, the scope follows along with the shaver, from the MCU portal, over the ganglion, toward the MCU portal, and finally into the joint. This is mainly to see the cyst wall (15). The shaver is always in the MCR position but is extraarticular. The MCU portal has limited vision and poor maneuverability, and the shaver cannot be completely observed from all angles. This increases the difficulty of the operation and the possibility of complications.

Entering from the complete midcarpal dorsal portal, the peripheral vision of the dorsal ganglion is incomplete and the range of motion of the arthroscope is small. Moreover, the visual field during the operation is limited, and the surrounding tissue of the cannula can easily be damaged when the arthroscope is operated from a large angle.

If the midcarpal volar portal is established, the medial scaphoid curve and inferior portion of the scapholunate joint can be seen in a better view (13). The dorsal ganglion has complete peripheral vision, and arthroscopy has a wide range of motion and does not require a large angle to operate. However, mirroring operation requires a certain learning curve of proficiency.

Some people have thick palms, and our trocar cannula is too short, making it difficult to establish a volar portal by the Kiss-in method. The long cannula is more suitable for the Kiss-in method. The volar portal increases the accessory injury to nerves, blood vessels, and tendons. The establishment of volar portal safety can be the problem requiring further discussion. In our study, 12 cases of dorsal ganglia had excellent results without accessory injury, suggesting that the Kiss-in method may represent a better way to solve the problem.

However, our series remains relatively restricted, which implies that our experimental evidence is not enough. We only have the PRWE scores for the patients. Due to small numbers, effects on important clinical outcomes could not be adequately assessed. Sample size expansion will be a future goal.

## Conclusions

The establishment of a midcarpal volar portal is safe by the Kiss-in method. The operative field is enlarged, and the volar portal visual field is better for the operator. Unlike the “inside-out” method of the mandrel, the Kiss-in method requires a vision or an extra portal to establish the volar portal. Incisions are easily reduced in the volar portal.

The establishment of a midcarpal volar portal for dorsal ganglion resection is a promising approach. It has a better visual field and a larger range of motion than the arthroscope. There are two incisions, volar and dorsal, respectively, allowing achieving an esthetic appearance.

## Data Availability

The original contributions presented in the study are included in the article/Supplementary Material, further inquiries can be directed to the corresponding author/s.
